# Coordination Chemistry of Uranyl Ions with Surface-Immobilized Peptides: An XPS Study

**DOI:** 10.3390/molecules27248960

**Published:** 2022-12-16

**Authors:** Esha Mishra, Cody M. Schultz, Rebecca Y. Lai, Peter A. Dowben

**Affiliations:** 1 Department of Physics and Astronomy, Theodore Jorgensen Hall, 855 North 16th Street, University of Nebraska-Lincoln, Lincoln, NE 68588-0299, USA; 2 Department of Chemistry, Hamilton Hall, University of Nebraska-Lincoln, Lincoln, NE 68588-0304, USA

**Keywords:** uranium detection, biosensor, XPS

## Abstract

The coordination chemistry of uranyl ions with surface immobilized peptides was studied using X-ray photoemission spectroscopy (XPS). All the peptides in the study were modified using a six-carbon alkanethiol as a linker on a gold substrate with methylene blue as the redox label. The X-ray photoemission spectra reveal that each modified peptide interacts differently with the uranyl ion. For all the modified peptides, the XPS spectra were taken in both the absence and presence of the uranium, and their comparison reveals that the interaction depends on the chemical group present in the peptides. The XPS results show that, among all the modified peptides in the current study, the (arginine)_9_ (R9) modified peptide showed the largest response to uranium. In the order of response to uranium, the second largest response was shown by the modified (arginine)_6_ (R6) peptide followed by the modified (lysine)_6_ (K6) peptide. Other modified peptides, (alanine)_6_ (A6), (glutamic acid)_6_ (E6) and (serine)_6_ (S6), did not show any response to uranium.

## 1. Introduction

Uranium is a heavy element naturally occurring and existing in the environment in various forms. The uranium occurring naturally is composed mainly of three radioactive isotopes, U-238 (99.3%), U-235 (0.7%) and U-234 (0.005%), the percentages denoting total composition by weight [1]. Uranium is commonly found in soil, rocks and groundwater, with concentrations depending upon the geological formation [2]. As groundwater is one of the major sources of drinking water in many parts of the world, uranium-contaminated water poses a serious health risk, as uranium is detrimental to biological systems due to its chemical toxicity [3]. The uranium in water and soils is frequently present as the soluble uranyl ion, or U(VI), that can react with biological molecules and give rise to various toxicological effects resulting in health hazards [4,5,6,7,8,9]. Secondary contamination of uranium in groundwater can be enhanced due to the presence of nitrates in the soil from chemical and organic fertilizers which undergo biological reduction, but also can enhance the solubility of uranium in groundwater [10]. Other major sources of uranium accumulation in the environment are due to nuclear weapons testing, ore processing, nuclear waste and, more recently, because of the use of depleted uranium in projectiles [11].

This wide prevalence of heavy metal toxins like uranium has encouraged the development of sensors for the detection of different metal ion contaminations. Different types of biosensors based on enzymes, DNA, proteins and peptides have been developed and studied [12,13,14,15,16]. An electrochemical peptide-based sensor derived from a peptide sequence of a Ni (II)-dependent NikR protein was studied as a possible uranyl ion sensor which could successfully recognize U(VI) [17]. The ability of peptides to form a robust assembly, which can be accompanied by molecular recognition features, are among the important aspects of peptide-based biosensors [18]. The multidentate peptide structure can have multiple coordination sites for the target metal ion along the peptide backbone [19]. 

**Figure 1:**
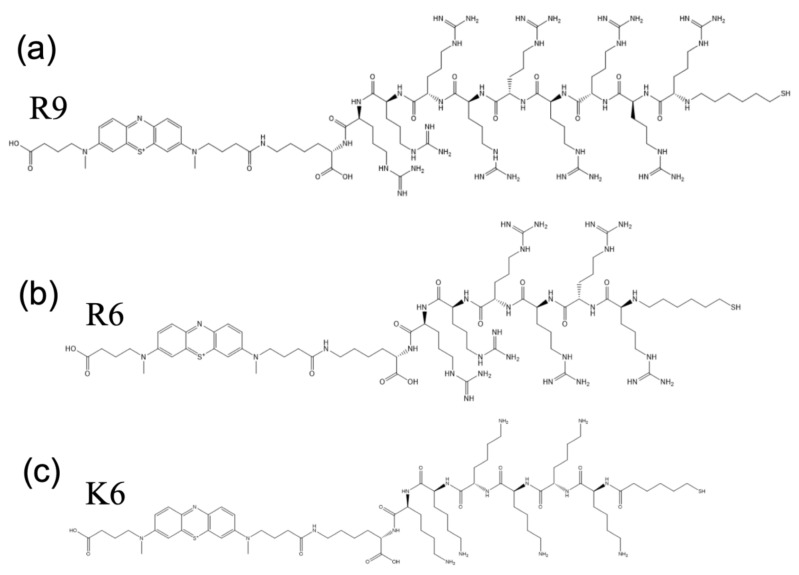
(**a**–**c**) represent the schematic chemical structure of the modified peptides; (**a**) (arginine)_9_ (R9), (**b**) (arginine)_6_ (R6) and (**c**) (lysine)_6_ (K6).

The interactions between U (VI) and various proteins, including albumin, fetuin and calmodulin, have been studied in recent decades [20,21]. Similarly, its interactions with specific amino acids have also been investigated [22,23,24,25]. However, most studies only focused on the interactions between U (VI) and single amino acids or protein-derived peptides, instead of homo-polypeptides such as the six sequences used in the current work. Although longer peptide sequences will provide more binding sites for U (VI), such polypeptides are not ideal for use with surface-based sensors, including the current thiol-gold self-assembled monolayer-based sensors. Depending on the specific sequences, longer peptides (e.g., 12+ amino acids) might not immobilize properly on the electrode, resulting in sensors with lower probe coverages. A relatively high probe coverage (e.g., 1 × 10^11^ molecules/cm^2^ or higher) is needed for U(VI) detection and in general for monolayer stability. Sensors with a low probe coverage are less stable, which is not ideal for sensing applications [26]. Furthermore, if the probe coverage is too low, the dynamic range of the sensor will also be affected. Thus, we chose six-amino acid as the standard probe length for this work. However, we did include a nine-amino acid sequence, (arginine)_9_ or R9 (see Figure 1), in this study. R9 was included because the (arginine)_6_ (R6) polypeptide showed reasonable interactions with uranium oxide ions, so we were interested in assessing whether a longer sequence would show better sensor response (i.e., higher % signal suppression).

In the past, traditionally the characterization of heavy metal biosensors has relied on electrochemistry techniques [15,16,17]; however, in this paper, we have described the interaction of uranyl ions with surface immobilized peptides using X-ray photoemission spectroscopy (XPS) in combination with electrochemistry techniques. The use of X-ray photoemission spectroscopy (XPS) is not unprecedented as XPS has been commonly used in the study of amino acid–metal interactions, peptide–metal interactions and DNA–heavy metal ion interactions [27,28,29]. The use of six surface immobilized peptides, (alanine)_6_ (A6), (glutamic acid)_6_ (E6), (arginine)_9_ (R9), (arginine)_6_ (R6), (serine)_6_ (S6) and (lysine)_6_ (K6), with and without exposure to U(VI) were studied.

## 2. Results and Discussion

### 2.1. Evidence of (UO_2_) Uptake from X-ray Photoemission

**Figure 2:**
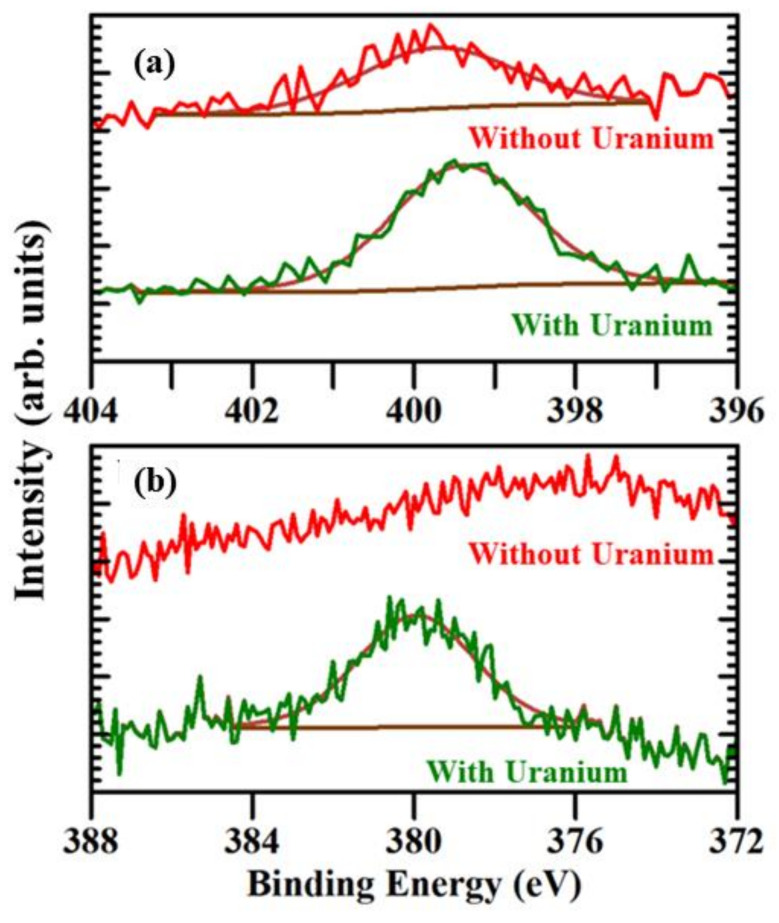
The XPS core level spectra of (**a**) N 1s and (**b**) U 4f_7/2_ for modified (arginine)_9_ peptide without and with soluble uranyl exposure.

Figure 2a,b shows the core level XPS spectra of N 1s and U 4f_7/2_, respectively, of modified (arginine)_9_ peptide with and without soluble uranyl exposure. As shown in Figure 2a, for the modified (arginine)_9_ peptide not exposed to uranium, the N 1s peak is at a binding energy of around 399.8 ± 0.3 eV; however, for the modified (arginine)_9_ peptide exposed to uranium, the N 1s peak is observed at a binding energy of 399.5 ± 0.2 eV. The back charge donation from the uranium oxide to the polypeptide backbone is significant enough to cause the shift of about 300 meV in the nitrogen core level binding energy. Figure 2b shows the comparison of the XPS core level spectra for uranium U 4f_7/2_ of the modified (arginine)_9_ peptide not exposed to soluble uranium oxide ions and modified (arginine)_9_ peptide exposed to soluble uranium oxide ions. For the modified (arginine)_9_ peptide not exposed to uranium, the U 4f_7/2_ peak is absent; however, for the modified (arginine)_9_ peptide exposed to soluble uranium oxide ions, the U 4f_7/2_ peak is observed at a binding energy around 380 ± 0.6 eV, which is in general agreement with the binding energies reported in the literature [30,31,32]. Each arginine has an additional guanidino group from the side chain, and because of this, the modified (arginine)_9_ peptide contains nine guanidino groups. Among the other basic compounds of nitrogen, specifically molecules with N linked to carbon such as amines and pyridines, guanidine has the strongest basicity [33]. The delocalization of the positive charge upon protonation when the guanidinium cation is formed is one of the reasons that guanidine has strong basic characteristics [34]. Guanidine can also function as a ligand upon metal complex formation, possibly enhanced as a result of delocalization due to the presence of lone pairs in nitrogen in the guanidine [34]. The presence of nine guanidino groups increases the number of coordination sites for the interaction of the uranyl ion with the modified (arginine)_9_ peptide, resulting a strong U 4f_7/2_ core level XPS peak, as shown in Figure 2b.

**Figure 3:**
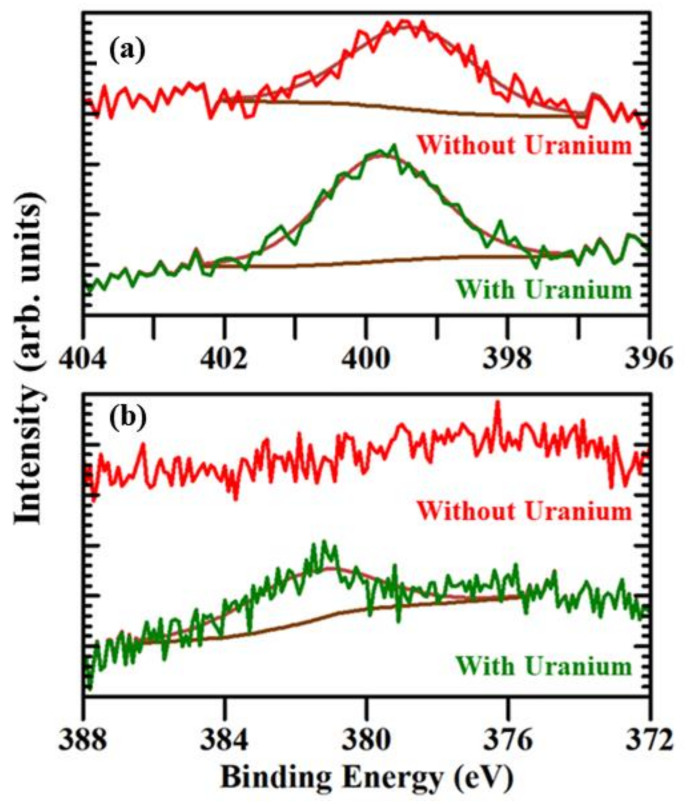
The XPS core level spectra of (**a**) N 1s and (**b**) U 4f_7/2_ for modified (arginine)_6_ peptide without and with soluble uranyl exposure.

Figure 3a,b shows the core level XPS spectra of N 1s and U 4f_7/2_, respectively, of the modified (arginine)_6_ peptide with and without soluble uranyl exposure. As shown in Figure 3a, for the modified (arginine)_6_ peptide not exposed to uranium, the N 1s peak is observed at a binding energy of around 399.2 ± 0.2 eV; however, for the modified (arginine)_6_ peptide exposed to uranium, the N 1s peak is observed at around 399.6 ± 0.2 eV. The shift of N 1s peak towards the higher binding energy for the modified (arginine)_6_ peptide exposed to uranium suggests that there is the charge transfer from the (arginine)_6_, i.e., R6, peptide to uranium oxide, but no back charge donation from the uranium oxide to the (arginine)_6_ peptide. Figure 3b shows the comparison of the XPS core level spectra for uranium (IV) U 4f_7/2_ of the modified (arginine)_6_ peptide not exposed to uranium and modified (arginine)_6_ peptide exposed to uranium. For the modified (arginine)_6_ peptide not exposed to soluble uranium oxide ions, the U 4f_7/2_ peak is absent; however, for the modified (arginine)_6_ peptide exposed to uranium, the U 4f_7/2_ peak is observed at a binding energy of around 381.2 ± 0.5 eV, suggesting an interaction between the uranyl (VI) and modified (arginine)_6_ peptide. The U 4f_7/2_ XPS core level peak area of the modified (arginine)_6_ peptide is smaller in comparison to the modified (arginine)_9_ peptide as the number of guanidino groups in the modified (arginine)_6_ peptide is less than the modified (arginine)_9_ peptide, which potentially leads to the reduction of the number of metal complexation sites for soluble uranium oxide ions.

**Figure 4:**
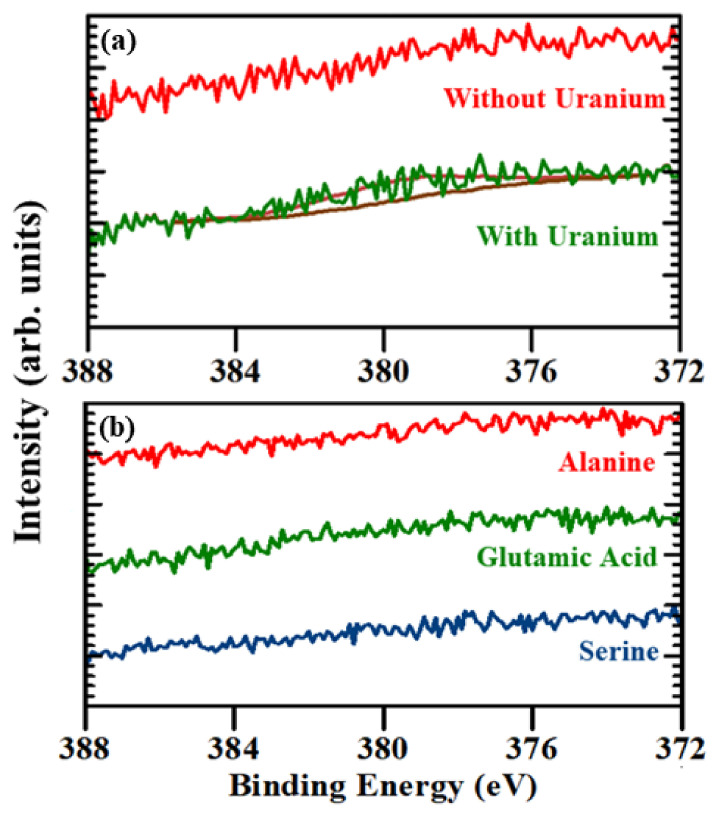
The XPS core level spectra of (**a**) the U 4f_7/2_ for (lysine)_6_ modified peptide with and without soluble uranyl exposure and (**b**) the U 4f_7/2_ for the (alanine)_6_, (glutamic acid)_6_ and (serine)_6_ modified peptides following soluble uranyl exposure.

Other modified peptides, (alanine)_6_ (A6), (glutamic acid)_6_ (E6) and (serine)_6_ (S6), schematically shown in the Supplementary Materials, did not show any response to uranium. Figure 4a shows the comparison between the XPS core level spectra for uranium U 4f_7/2_ of the (lysine)_6_ modified peptide not exposed to soluble uranium oxide ions and the (lysine)_6_ modified peptide exposed to soluble uranium oxide ions. For the (lysine)_6_ modified peptide not exposed to uranium, the U 4f_7/2_ peak is absent; however, for the (lysine)_6_ modified peptide exposed to uranium, the U 4f_7/2_ XPS core level peak is barely observed at around 379.5 ± 4.0 eV, suggesting there is some interaction of uranium with the (lysine)_6_ modified peptide. Better evidence of soluble uranyl exposure arises from the (lysine)_6_ modified peptide electrochemical studies discussed below. If we compare the U4f_7/2_ XPS core level spectra of the (lysine)_6_ modified peptide with the U 4f_7/2_ spectra of the (arginine)_9_ and (arginine)_6_ modified peptide, the U 4f_7/2_ core level XPS peak area is smallest in the (lysine)_6_ modified peptide, suggesting that the interaction between uranium and the (lysine)_6_ modified peptide is not as strong as the interaction of uranium with the (arginine)_9_ and (arginine)_6_ modified peptides. Each lysine has an additional -NH_2_ group from the side chain and the (lysine)_6_ modified peptide contains six -NH_2_ group in the peptide backbone. The lower basic strength of the -NH_2_ group in comparison to the guanidino group potentially decreases the U 4f_7/2_ XPS core level peak area. Figure 4b shows the comparison of the XPS core level spectra for uranium U 4f_7/2_ of the modified (alanine)_6_, (glutamic acid)_6_ and (serine)_6_ peptides exposed to uranium. Although the other modified peptides, (alanine)_6_ (A6), (glutamic acid)_6_ (E6) and (serine)_6_ (S6), also have a nitrogen containing amide group in their peptide backbone, the amides are even less basic than amines and the lone pair of electrons in the nitrogen of amide group are delocalized during resonance, resulting in no significant response to the presence of uranium. As shown in Figure 4b, for all three (alanine)_6_, (glutamic acid)_6_ and (serine)_6_ modified peptides exposed to uranium, the U 4f_7/2_ peak is absent in the XPS spectra, suggesting no significant interaction between the uranium and the respective polypeptides. Additionally, the U 4f_7/2_ XPS core level spectra for the control experiments (HS-C6-K-MB) and (HS-C6-OH) in the Supplementary Material Figures S1 and S2, respectively, suggest that there is no significant uranium interaction with (HS-C6-K-MB) and (HS-C6-OH), where MB is methylene blue.

### 2.2. Modified Polypeptides as Sensors Characterization

**Figure 5:**
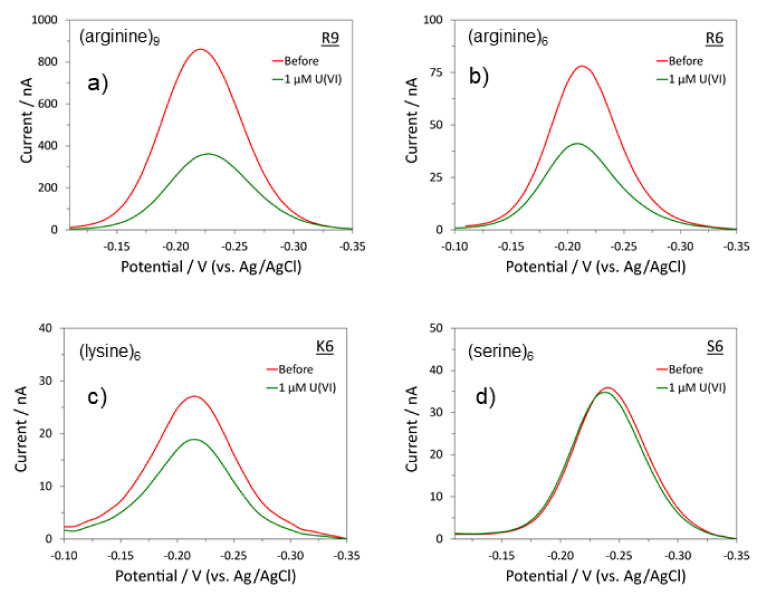
The alternating current voltammetry (ACV) curves of (**a**) (arginine)_9_ modified peptide, (**b**) (arginine)_6_ modified peptide, (**c**) (lysine)_6_ modified peptide and (**d**) (serine)_6_ modified peptide before and after the addition of 1 µM U(VI).

The characterization of the modified polypeptides as sensors is seen to be consistent with the XPS investigations of the heavy metal soluble (UO_2_) ion interactions. Electrochemically, we determined the apparent coverages using the methylene blue (MB) signal after the equilibration step (Table 1). The peptide concentration used in the sensor fabrication step was optimized to achieve the highest possible probe coverage without causing major instability in the monolayer. As shown in Figure 5, if we compare the coverage of three modified peptides, i.e., (arginine)_9_, (arginine)_6_ and (lysine)_6_, the highest coverage is in the order: (arginine)_9_ modified peptide > (arginine)_6_ modified peptide > (lysine)_6_ modified peptide, which is consistent with the XPS results presented above. The peptides with the highest and lowest probe coverages were the (arginine)_9_ modified peptide and the (serine)_6_ modified peptide, respectively. Overall, there was no correlation between coverage and interaction with uranium, further suggesting that the probe identity was the most influential portion of interaction.

The limits of detection (LODs) for (arginine)_9_ (R9), (b) (arginine)_6_ (R6) and (c) (lysine)_6_ (K6) based on the electrochemical sensors fabricated on gold disk electrodes are R6: 10 nM, R9: 10 nM and K6: 100 nM. The LODs for R6 and R9 are five times lower than the two U(VI) sensors we developed previously [17,24]. We did not obtain LODs for A6, S6 and E6 since they did not show any substantial interactions with U(VI).

## 3. Materials and Methods

Sodium hydroxide, 6mercapto1-hexanol (C6-OH), hydrogen peroxide, sulfuric acid, sodium chloride (NaCl), potassium chloride (KCl), acetonitrile (ACN), dimethyl sulfoxide (DMSO), Trizma-base and Tris-HCl were used as received (Sigma-Aldrich, St. Louis, MO, USA). Depleted uranium (VI) (UO_2_(NO_3_)_2_) standard was purchased from Ricca Chemical Company. Deionized (DI) water was purified using a Synergy Ultrapure Water System (18.2 MΩ·cm, Millipore, Billerica, MA, USA) and was used to make all solutions and dilutions in this study.

The modified peptides used in our study are a self-assembled monolayer with a 6-carbon alkanethiol as a linker and methylene blue (MB) as a redox label on gold substrate (Xaia Peptides, Inc., Göttenberg, Sweden). The six modified peptides were (i) an alanine (A6) modified peptide: HS-C6-(alanine)_6_-K-MB, (ii) a glutamic acid (E6) modified peptide: HS-C6-(glutamic acid)_6_-K-MB, (iii) an arginine (R6) modified peptide: HS-C6-(arginine)_6_-K-MB, (iv) a longer arginine (R9) modified peptide: HS-C6-(arginine)_9_-K-MB, (v) a serine (S6) modified peptide: HS-C6-(serine)_6_-K-MB and (vi) a lysine (K6) modified peptide: HS-C6-(lysine)_6_-K-MB.

R6, E6, A6 and S6 were reconstituted in 50% ACN and 50% DI water, K6 and R9 were reconstituted in DI water and K-MB was reconstituted in 43% ACN, 13.5% DMSO and 43.5% DI water. All except K-MB were reconstituted to 250 µM while K-MB was reconstituted to 630 µM. All were diluted to the appropriate concentrations for sensor fabrication (R6: 10 µM, R9: 10 µM, K6: 20 µM, A6: 10 µM, S6: 5 µM, E6: 15 µM and K-MB: 1 µM). The sensor interrogation buffer was a pH 7.3 Phys2 buffer which consisted of 18.4 mM Tris-HCl, 1.5 mM Trizma-base, 140 mM NaCl and 5 mM KCl.

Gold-coated glass (1000 Å gold with 50 Å titanium binding layer) substrates (chips) were prepared by cutting the slides into ~1 cm × ~1 cm chips. The chips were then treated with piranha solution (concentrated sulfuric acid with 30% hydrogen peroxide 3:1) for 10 s to clean the gold. The chips were then further cleaned by UV/ozone treatment. For the electrochemical measurements, the gold was electrochemically cleaned by repetitive oxidation and reduction in 0.5 M H_2_SO_4_ from −0.3 V to +1.5 V (vs. Ag/AgCl). The real surface area was determined in 0.05 M H_2_SO_4_ using the reduction peak of gold oxide at ~0.85 V using the reported value of 400 µC/cm^2^. The ratio between the real and geometric areas was used to calculate the surface roughness or roughness factor. Gold chips with roughness factors of 1–1.4 were used for further modification.

All samples were exposed to the modified peptide for 1 h at 4 °C followed by 16–18 h of 2 mM C6-OH at 4 °C. The samples were then rinsed with DI water and equilibrated for 1 h in a Phys2 buffer. 1 µM of U(VI) was then added to the buffer and allowed to equilibrate with each sample for an additional hour.

An Al-Kα X-ray source (photon energy, 1486.6 eV) with a hemispherical electron energy analyzer in an ultrahigh vacuum (UHV) system was used to acquire the XPS spectra. The VG-100, a VG Hemispherical analyzer with 50 eV pass energy, was used for all XPS measurements. CASAXPS software was used to analyze all the core level XPS spectra and Shirley background was employed for the background correction. To account for the charging effect, the binding energies were referenced to the C 1s carbon peak at 284.7 eV. The X-ray spot size was approximately 0.75 cm across and is more suitable for these measurements compared to a more focused beam spot size, as the lower flux density diminishes X-ray and secondary electron damage to the polypeptides. No charge neutralization was used in our measurements to minimize degradation to the samples. The typical system pressure during the analysis was approximately 1 × 10^−9^ torr and the photoemission take off angle was along the surface normal. The typical sampling depth is difficult to know without precise calibration for this density of polypeptides; however, based on the previous studies where the photoelectron mean free path was through the self-assembled organic monolayer systems, the mean free path was about 28–42 Å [35]. The addition of UO_2_ to the polypeptide samples likely shrunk this mean free path.

Electrochemical measurements were performed with a CH Instruments 1040A potentiostat (Austin, TX, USA). A conventional three-electrode system consisting of a polycrystalline gold disk working electrode (area approx. 0.0314 cm^2^), an Ag/AgCl (1 M KCl) reference electrode (CH Instruments, Austin, TX, USA) and a platinum counter electrode was used. Alternating current voltammetry (ACV) at 10 Hz was used to characterize the sensors during the stability assessment and target interrogation. Lower frequencies (1–4 Hz) were used to calculate the peptide probe coverage using the following equation:

$$I_{\mathrm{avg}}\left(E^{0\prime }\right)=2nfFN_{tot}\frac{\sin h\left(nFE_{ac}/RT\right)}{\cos h(nFE_{ac}/RT)+1}$$

where I_avg_($E^{0\prime }$) is the average AC peak current in the voltammogram, *n* is the number of electrons transferred (MB = 2), *F* is the Faraday constant, *R* is the universal gas constant, *T* is the temperature, *E_ac_* is the peak amplitude and *f* is the frequency of the AC voltage [36,37]. The value of *N_tot_* for each frequency was averaged and divided by the electrode area to obtain the probe coverage.

The MB peak current was monitored in real time with ACV at 10 Hz during the equilibration step. The % signal suppression (%*SS*) in the presence of U(VI) was determined using the following equation:

$$\%SS=\frac{I_{0}-I}{I_{0}}\times 100\%$$

where *I* is the baseline-subtracted MB peak current after addition of U(VI) and *I_0_* is the baseline-subtracted MB peak current in the interrogation buffer without U(VI). The addition of the MB redox label enables direct electrochemical characterization of the peptides during both equilibration and target interaction steps [17]. The MB current was also used to determine the probe coverage for each peptide. Without the MB label, the amount of peptide incorporated into the monolayer could not be verified electrochemically.

## 4. Conclusions

In this study, we have investigated the interactions of uranyl ions with six different surface immobilized peptides using XPS and electrochemical measurements. The XPS analysis shows that the modified arginine peptides, (arginine)_9_ and (arginine)_6_, and the lysine modified peptide, (lysine)_6_, are capable of detecting uranium, with the (arginine)_9_ modified peptide demonstrating the largest response to uranium. The short peptide length of (arginine)_6_ compared to (arginine)_9_ reduces the coordination sites for uranium to interact with the guanidino group present in the arginine side chain, resulting in the uranium response being smaller than the (arginine)_9_ modified peptide. The (lysine)_6_ modified peptide does not have guanidino groups in its side chain, it instead has -NH_2_ groups, which are less basic in comparison to guanidino groups, and hence it shows a limited response in comparison to (arginine)_9_ and (arginine)_6_ modified peptides. The other modified peptides, (alanine)_6_, (glutamic acid)_6_ and (serine)_6_, did not demonstrate any response to uranium. These findings illustrate that the side chains of the amino acid play an important role in binding with uranium.

## Figures and Tables

**Table 1:** Peptide probe coverages determined from the methylene blue (MB) peak currents after sensor equilibration using electrochemical techniques.

Probe	Coverage (Teramolecules/cm^2^)
(arginine)_6_ (R6)	1.37
(arginine)_9_ (R9)	8.89
(lysine)_6_ (K6)	0.15
(alanine)_6_ (A6)	9.20
(serine)_6_ (S6)	0.55
(glutamic acid)_6_ (E6)	1.70
K-MB	1.23

## Data Availability

The Supplementary Materials contains data supporting the reported results.

## Supplementary Materials

The following supporting information can be downloaded at: https://www.mdpi.com/article/10.3390/molecules27248960/s1, Figure S1: The U 4f_7/2_ XPS core level spectra for the control experiment (HS-C6-K-MB); Figure S2: The U 4f_7/2_ XPS core level spectra for the control experiment (HS-C6-OH); Figure S3: The schematic chemical structure of the modified peptides (alanine)_6_ (A6), (glutamic acid)_6_ (E6) and (serine)_6_ (S6).
