# Supplementary material for: Coordination Chemistry of Uranyl Ions with Surface-Immobilized Peptides: An XPS Study
Source: Molecules. 2022 Dec 16;27(24):8960. doi: 10.3390/molecules27248960 (PMC9784848; doi:10.3390/molecules27248960)
Supplement: Supplementary file 1 [file molecules-27-08960-s001.zip › molecules-2045540-supplementary.pdf]

Supplementary Information

Coordination Chemistry of Uranyl Ions with Surface-Immobilized Peptides: An XPS Study

Esha Mishra [1], Cody M. Schultz [2], Rebecca Y. Lai [2] and Peter A. Dowben [1,*]

[1] Department of Physics and Astronomy, Theodore Jorgensen Hall, 855 North 16th Street, University of Nebraska-Lincoln, Lincoln, NE 68588-0299, USA

[2] Department of Chemistry, Hamilton Hall, University of Nebraska-Lincoln, Lincoln, NE 68588-0304, USA

* Correspondence: pdowben@unl.edu

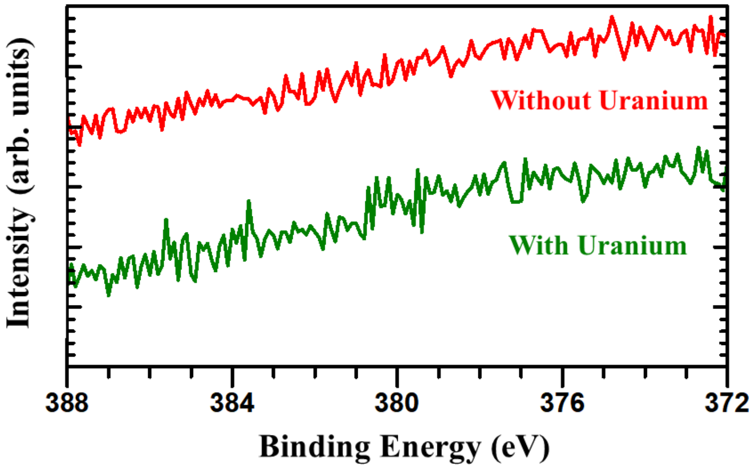

Figure S1: The U $4f_{7/2}$ XPS core level spectra for the control experiment (HS-C6-K-MB), where MB is methylene blue.

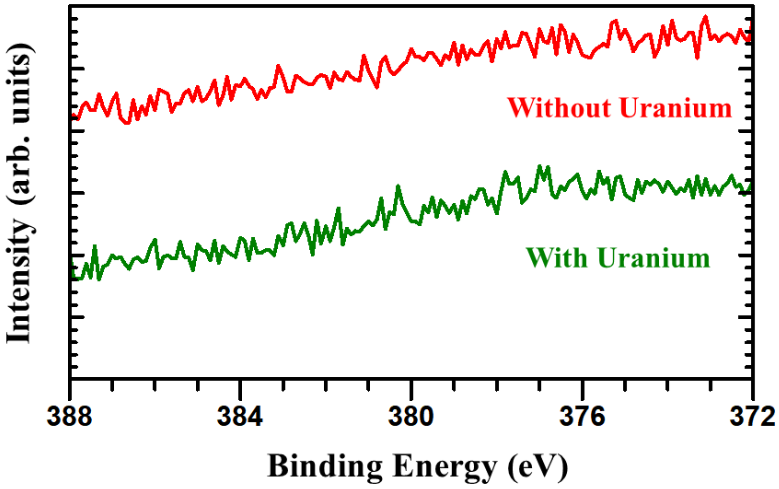

Figure S2: The U $4f_{7/2}$ XPS core level spectra for the control experiment (HS-C6-OH)

A6

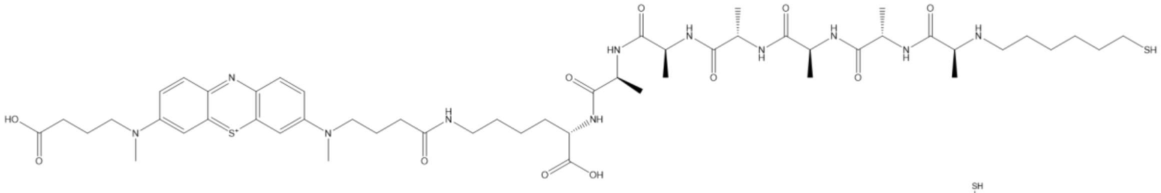

E6

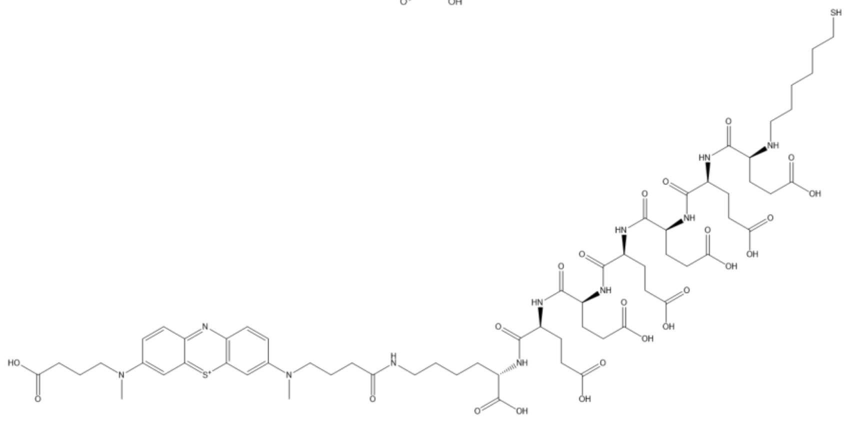

S6

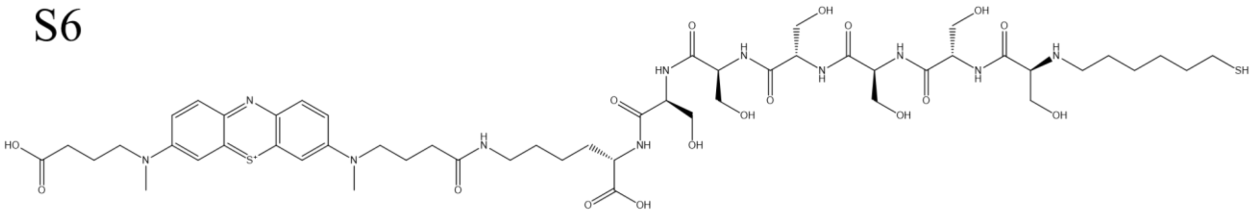

Figure S3: The schematic chemical structure of the modified peptides (alanine)$_6$ (A6), (glutamic acid)$_6$ (E6) and (serine)$_6$ (S6).
